# Coupling of shoulder joint torques in individuals with chronic stroke mirrors controls, with additional non-load-dependent negative effects in a combined-torque task

**DOI:** 10.1186/s12984-021-00924-1

**Published:** 2021-09-08

**Authors:** Joseph V. Kopke, Levi J. Hargrove, Michael D. Ellis

**Affiliations:** 1 Department of Biomedical Engineering, McCormick School of Engineering, Northwestern University, 2145 N Sheridan Rd, Evanston, IL 60208 USA; 2 Center for Bionic Medicine, Shirley Ryan AbilityLab, 355 East Erie, Chicago, IL 60611 USA; 3 Department of Physical Therapy and Human Movement Sciences, Northwestern University, 645 N Michigan Ave, Suite 1100, Chicago, IL 60611 USA

**Keywords:** Stroke, Hemiparesis, Synergy, Shoulder, Strength, Rotation, Weakness

## Abstract

**Background:**

After stroke, motor control is often negatively affected, leaving survivors with less muscle strength and coordination, increased tone, and abnormal synergies (coupled joint movements) in their affected upper extremity. Humeral internal and external rotation have been included in definitions of abnormal synergy but have yet to be studied in-depth.

**Objective:**

Determine the ability to generate internal and external rotation torque under different shoulder abduction and adduction loads in persons with chronic stroke (paretic and non-paretic arm) and uninjured controls.

**Methods:**

24 participants, 12 with impairments after stroke and 12 controls, completed this study. A robotic device controlled abduction and adduction loading to 0, 25, and 50% of maximum strength in each direction. Once established against the vertical load, each participant generated maximum internal and external rotation torque in a dual-task paradigm. Four linear mixed-effects models tested the effect of group (control, non-paretic, and paretic), load (0, 25, 50% adduction or abduction), and their interaction on task performance; one model was created for each combination of dual-task directions (external or internal rotation during abduction or adduction). The protocol was then modeled using OpenSim to understand and explain the role of biomechanical (muscle action) constraints on task performance.

**Results:**

Group was significant in all task combinations. Paretic arms were less able to generate internal and external rotation during abduction and adduction, respectively. There was a significant effect of load in three of four load/task combinations for all groups. Load-level and group interactions were not significant, indicating that abduction and adduction loading affected each group in a similar manner. OpenSim musculoskeletal modeling mirrored the experimental results of control and non-paretic arms and also, when adjusted for weakness, paretic arm performance. Simulations incorporating increased co-activation mirrored the drop in performance observed across all dual-tasks in paretic arms.

**Conclusion:**

Common biomechanical constraints (muscle actions) explain limitations in external and internal rotation strength during adduction and abduction dual-tasks, respectively. Additional non-load-dependent effects such as increased antagonist co-activation (hypertonia) may cause the observed decreased performance in individuals with stroke. The inclusion of external rotation in flexion synergy and of internal rotation in extension synergy may be over-simplifications.

## Background

Approximately 610,000 new strokes occur each year in the US and 16.9 million occur worldwide [1]. Currently, 6.6 million Americans are living post stroke, approximately 30–60% of whom have chronic upper extremity motor impairments [2, 3], including weakness, loss of multi-joint coordination, hypertonia, and spasticity. Weakness and loss of multi-joint coordination involving the upper extremity may affect activities of daily living that require control of arm position, stiffness, damping, and inertia [4] to enable the individual to accomplish tasks such as feeding themselves, dressing, preparing food, carrying objects, or opening doors.

One factor that contributes to the loss of multi-joint coordination after stroke is an unintentional co-contraction of muscles throughout a limb, described as an abnormal synergy, and loss of independent joint control [5–7]. Shoulder abduction is reported as being accompanied by shoulder external rotation, elbow flexion, supination, and wrist and finger flexion, while shoulder adduction is often accompanied by shoulder internal rotation, elbow extension, and wrist and finger flexion [8].

Using an isometric task in single directions, Dewald et al. compared control, non-paretic, and paretic internal and external rotation torques generated during shoulder abduction and found inconsistencies with the expectations of the abnormal synergy hypothesis [9]. Specifically, the paretic and control arms had similar secondary torque generation patterns in internal and external rotation that were different from the non-paretic arms. This result was not investigated further, the focus of subsequent work being on the more robust effects of abductor drive on distal joints, including elbow, wrist, and fingers [10].

We have also moved away from analyzing secondary torques (torques generated in directions that participants are not instructed to control or do not have feedback from) during single direction tasks because of the difficulty in determining if those torques are pathological (mandatory), normal, or just how these individuals chose (consciously or unconsciously) to perform the task [7, 11]. We have instead moved towards multi-degree of freedom (DOF) tasks that test ability in two or more directions simultaneously [12, 13]. At the cost of minimally increasing cognitive load [14], these tasks allow us to better test movement capacity after stroke, and to determine whether it is limited by neural or mechanical constraints. Importantly, this dual-task paradigm has been completed for multi-joint combinations of shoulder abduction, elbow extension, and wrist/finger extension [9, 11, 15] but not for within-shoulder movements such as internal and external rotation during abduction or adduction. Furthermore, our recent work has suggested that internal/external rotation and abduction/adduction joint torque coupling may be present in individuals without stroke. These results warrant an in-depth analysis of shoulder DOF coupling in individuals with and without stroke.

Minimal work has been published on attempts to quantify torque generation capacity at the shoulder (glenohumeral joint) during multi-DOF tasks. Baillargeon et al. recently published a study examining feasible torque space of the shoulder in young healthy adults [16]. They noted that external rotation during adduction and internal rotation during abduction were the weakest directions of the shoulder. Although these data are from unimpaired individuals, these torque combinations are considered to be out-of-synergy in individuals with stroke. Beer et. al confirmed that hemiparetic external rotation weakness was profound (33%); however, this weakness was unrelated to reaching performance against gravity (movement out of synergy) [17]. Nonetheless, internal/external rotation capacity after stroke has largely been assumed to be constrained by abnormal neural drive.

This study attempts to better understand and quantify longstanding observations going back to the work of Twitchell et al. [18] that included external/internal rotation in flexion/extension synergies, respectively. The nature of external/internal rotation capacity is of special relevance because technically sophisticated devices and associated protocols are being designed for rehabilitation of the upper extremity [19]. This study examines internal and external rotation torque generation capacity (strength) during abduction and adduction using a robotic device—a paradigm similar to that first described by Beer et al. to investigate task-dependent weakness in elbow flexion/extension during abduction/adduction [20]. In line with the described clinical presentation and laboratory-based findings of multi-joint synergistic movement and posturing following a stroke, we hypothesized that, compared to individuals without stroke, the paretic arm would reflect abnormal synergy within the DOFs of the glenohumeral joint, such that external rotation would be weaker during adduction and stronger during abduction, and conversely that internal rotation would be stronger during adduction and weaker during abduction.

## Methods

Fourteen participants with chronic hemiparetic stroke and moderate to severe upper-extremity motor impairments were recruited and provided consent to participate. Moderate to severe motor impairment was determined, by a licensed physical therapist, as a score between 10 and 45 out of 66 on the upper-extremity portion of the Fugl-Myer Assessment. Two participants were excluded from analysis: one was not able to generate any external rotation torque and another was unable to execute the required dual-task. Thus, 12 participants (6 female) with hemiparetic stroke with chronic motor impairments (60.8 ± 10.3 years old, with a Fugl-Myer Upper-Extremity Assessment score of 26.9 ± 8.4, and 16.8 ± 8.3 years post-stroke) completed the study. Twelve age- and sex- matched participants without stroke (6 female, mean age 59.1 ± 9.9 years old) were also recruited to serve as controls. The non-paretic then the paretic arms were tested in those with stroke, while only the dominant arm was tested in control participants. All participants provided written consent to participate in the study in accordance with Northwestern University Institute Review Board (IRB #: STU00205835).

### Setup and instrumentation

Once seated in a rigid chair (Biodex, Shirley, NY; Model 830–110) with waist and shoulder straps fastened and secured, each participant’s arm was placed in 90° of abduction, 45° of horizontal adduction, and 90° of elbow flexion using a custom setup that put the arm in the transverse plane at shoulder height. The forearm was rigidly attached to the custom setup via a fiberglass cast that covered the hand, wrist, and forearm, enabling a 6-DOF load cell (JR3 Inc., Woodland, CA, USA; Model 45E15A) to measure 3-axis force and moment data.

### Single-DOF isometric strength

Isometric strength was collected via a single-DOF isometric task in four different directions in the following order: shoulder abduction, shoulder adduction, external rotation, and internal rotation. Five-second trials with verbal encouragement and visual feedback of real-time torque were repeated until three trials were collected in which the maximum torques in the testing direction were within 10% of each other and the maximum torque in the last trial was not the greatest. One-minute rest periods were provided between each trial. Load cell data and the corresponding anthropometrics for each participant were used to calculate maximal joint torques, a precision measurement of strength. The maximal adduction and abduction joint torque was used as input to the subsequent dual-task strength protocol.

### Dual-task strength setup

**Fig. 1:**
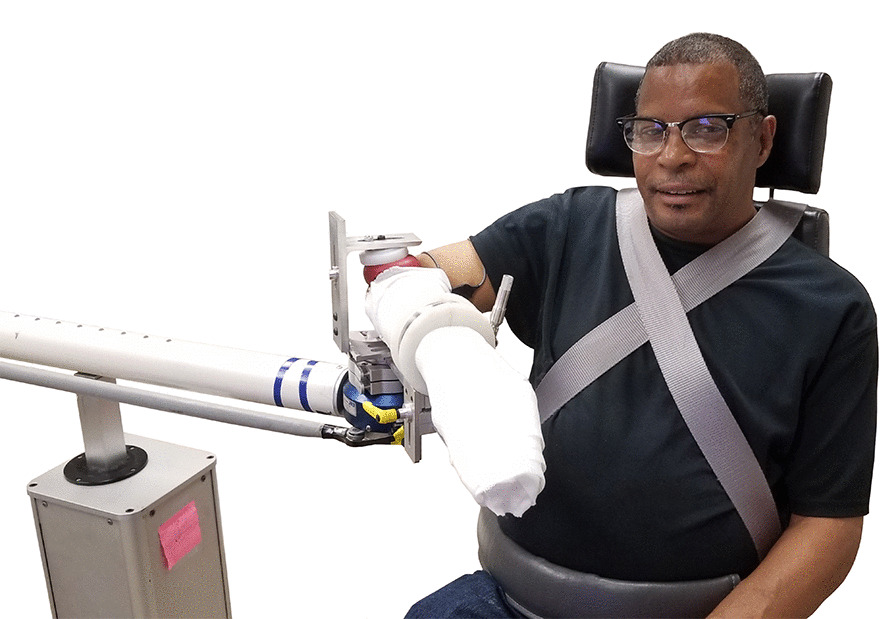
Dual-task setup. Custom ACT^3D^ with load cell (in blue under elbow) with casted arm attached via custom device. Movement of the robot was limited to the vertical direction. This participant’s arm is resting on lower horizontal surface

Participants moved from the isometric setup to a customized robotic setup that comprised a modified ACT^3D^ HapticMaster with an added 6-DOF load cell (JR3 Inc., Woodland, CA, USA; Model 51E20A) at the end effector. Each participant sat in a chair similar to that described for the isometric testing. Participants were rigidly connected to the load cell using a custom device that secured the medial and lateral epicondyles between foam pads centered over the load cell and attached the casted forearm to a rigid bar extending from the load cell, as depicted in Fig. 1. The elbow was centered over the load cell to minimize sensitivity to measurement error and to decouple the DOFs being tested. The position of the robot and the participant were adjusted so that the arm position was the same as for the isometric single-DOF strength testing (90° abduction, 45° horizontal adduction, and 90° elbow flexion).

### Dual-task strength testing

**Fig. 2:**
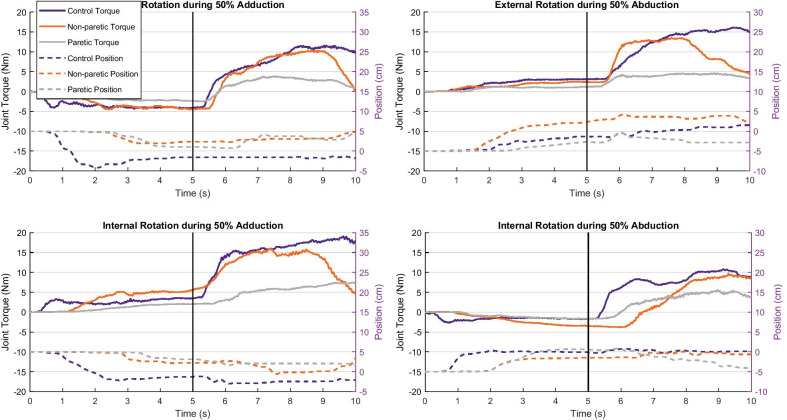
Representative trials for each lifting/humeral rotation direction combination for each group. Solid lines are the isometric humeral rotation joint torques (Nm; External Rotation (+) in the top panels, Internal Rotation (+) in the bottom panels) indicated by the left y-axis. Dashed lines are the vertical position of the robot (cm; 0 indicating midpoint between ceiling (+) and floor (−) limits) during the task indicated by the right y-axis. The starting position of the vertical position on the left two plots was 5 cm illustrating the participant beginning in contact with the ceiling limit. Similarly, the starting position of the vertical position on the right two plots was −5 cm illustrating the participant beginning in contact with the floor limit. The vertical black line indicates the transition cues (visual and verbal) from the single-task to the dual-task

Using the maximal abduction and adduction joint torque data from the isometric single-DOF strength testing, vertical loads (accounting for limb weight) were calculated and programmed into the device. These loads would require each participant to use 0, 25, or 50% of their maximal abduction or adduction strength to move the robot vertically depending on the condition being tested (5 loading conditions: 0, ± 25, ± 50%). An upper (5 cm above 90° of abduction) and lower (5 cm below 90° of abduction) vertical limit of joint position was created to control the extent of shoulder abduction or adduction. These somewhat arbitrary limits were set to ensure safety of the glenohumeral joint while enabling a more intuitive paradigm for controlling two DOFs simultaneously. Loads applied by the robot during adduction trials forced the at-rest limb toward the upper limit (ceiling) while abduction loads forced it toward the lower limit (floor). During each trial the participant was required to adduct or abduct against the specified load and maintain it within the 10 cm vertical window between the limits (quasi-static/dynamic). Each trial consisted of 5 s of single task abduction or adduction off of (within) the vertical limits followed by 5 s of maximal isometric internal or external rotation. Internal and external rotation torques that were generated while the limb made incidental contact with the upper or lower limits were not included in the analysis. A simple bar plot projected on a large monitor provided feedback of real-time calculated internal and external rotation joint torque. A visual and audible cue was given for both the start of the lift condition (first 5 s) and the lift plus rotation condition (last 5 s). We have found that a quasi-static/dynamic control of shoulder abduction and adduction position while under load alleviates the cognitive burden of a dual-task as it is more functionally intuitive than a fully isometric dual-task. Ten load levels were evaluated (5 for external rotation and 5 for internal rotation) for both the adduction and abduction conditions, and order of abduction/adduction loads and of internal and external rotation trials was randomized. Trials were repeated within each condition until three trials were acquired in which the maximal internal or external rotation torque was within 10% of each other and the last torque was not the greatest. Rest periods of one minute or longer were provided between each trial. Total trials needed to complete the protocol were similar for each group (Control: average = 43.4 trials (range 34–58), Non-paretic: 46.0 (36–57), Paretic: 42.6 (36–55)). Paretic arms were tested after non-paretic arms in attempt to minimize learning effects during testing of the paretic arm. Figure 2 shows representative trials for each combination of tasks (abduction or adduction paired with external or internal rotation) of one participant.

### Data processing

Load cell data were transformed into joint torques using anthropometric measurements and a series of coordinate transformations for both the single- and dual-tasks. All data were smoothed/averaged over 200 ms windows. The highest internal and external rotation torque was identified within each trial (torques acquired while contact was made with the ceiling or floor limits were ignored) and the largest torque for each condition and each participant was used for the analysis below. This resulted in one external rotation torque and one internal rotation torque for each of the 5 load levels (−50, −25, 0, 25, 50%), representing humeral rotation strength as a function of abduction/adduction load. Maximum torques were normalized to their maximum value attained during either the single- or dual-task to account for heterogeneous weakness/strength in the study cohort.

### Data analysis

Four linear mixed-effects models were generated from *normalized* dual-task strength data to test the effects of group, load-level, and their interaction on internal and external rotation strength (Eq. 1). Abduction and adduction data were separated as were external and internal rotation data. Each linear model was run within one task combination (e.g. external rotation during adduction). The 0% load condition was used in both the adduction as well as the abduction models resulting in four models with three load-levels (0, 25, and 50%) and three groups (C, NP, P). Sex was initially included as a main effect but was removed due to lack of significance in the testing of normalized dual-task strength and as it was not our primary parameter of interest. The models were formulated as follows with group and load as main factors and participant as a random factor:

1 $${\text{Rotation torque}} \sim group + load + group*load + (1|participant)$$

### OpenSim modeling

We simulated both the single- and dual-task protocols using musculoskeletal modeling via OpenSim with a previously validated model of the shoulder [21, 22]. The intent was to determine if model data reflected experimental data and offered additional insights into the musculoskeletal constraints of internal rotation/adduction coupling and external rotation/abduction coupling. Using the methods outlined previously [23, 24], we used linear programming to optimize muscle activations (in the range of 0–1) to maximize torque in particular directions within particular constraints or boundaries.

We matched the model posture to our experimental setup which equated to 40° “elevation angle”, 90° “shoulder elevation”, 50° “shoulder rotation”, and 90° of “elbow flexion”. Parameters for muscles crossing the shoulder were extracted and provided to customized optimization software written in MATLAB (Release 2017a, The MathWorks, Inc., Natick, MA, USA). Prior work showed that this model under-predicts the moment arm of the teres minor compared to values found via cadaveric testing, so we extracted values for the teres minor as well as for the other rotator cuff muscles from anatomical moment arm studies to better approximate the rotator cuff musculature [25, 26]. Additionally, the model is based on 50^th^ percentile male size and young male muscle volume. Muscle volume (peak force) was adjusted by multiplying each muscle by its ratio between an older adult male or female and the young male adult data that was used to generate the model [27, 28].

Hypothetical joint torque maximums (similar to single-DOF strength) were acquired by using the customized software to optimize muscle activations to maximize the joint torques in each of the single directions (abduction, adduction, external rotation, and internal rotation). Next, abduction and adduction loading was simulated by creating inequality constraints of abduction and adduction loads ranging between 0 and 100% ± 1% of the maximum torque. The optimization was then run to maximize internal and external rotation torque while simultaneously meeting the abduction or adduction loading constraints. This resulted in simulations of the described dual-task protocol across all possible load-levels.

To better simulate the weakness experienced after stroke and allow the model to generate torques closer to those achieved by our participants with stroke, all muscle forces were simulated as having strength that was 25%, 50%, or 75% of the maximal strength (Fig. 4*top and middle*). Each of these limits was applied across all 18 muscle segments included in the model including three deltoid segments, supraspinatus, infraspinatus, subscapularis, teres minor, teres major, three pectoralis major segments, three latissimus dorsi segments, coracobrachialis, the short and long head of the biceps and the long head of the triceps. The optimization was run again to maximize internal and external rotation torque under the prescribed strength and load constraints.

Finally, to investigate the effects and possible contribution of hypertonia on task performance, we imposed a lower bound on muscle activation. While the above simulations allowed for muscles to have no activation during the tasks (0%), these simulations required a minimum activation of 10 or 20% (Fig. 4. *bottom*) simulating increased background activity (hypertonia) across all muscles and tasks. These values were chosen as they would show the effect or trend of hypertonia within a reasonable range.

## Results

Single-DOF isometric strength values are summarized in Table 1 for all groups and directions presented as group mean (standard error) with N = 6 for each group and sex. Single-DOF OpenSim model data is also included in Table 1. Raw strength values were averaged by sex since we did not collect or control for muscle mass or cross-sectional area, and a known relationship exists between sex and muscle mass and between muscle mass and strength, resulting in sex-based differences in strength of 40 to 50% [29, 30]. Combined results for females and males is also provided to allow comparison with other published data that was not reported by sex. Relative strength ratios between each group provide a general indication of how strength varied between groups. Strength ratios presented in Table 1 are ratios of the group averages, since participants were not matched at the individual level. The strength of the paretic arm generally ranged between 25 and 50% of non-paretic arm and control arm strength.

**Table 1:** Single-DOF isometric strength data for each group and sex

	AB	AD	ER	IR
Female				
Control	39.6 (1.3)	38.9 (1.5)	19.4 (1.7)	16.6 (1.1)
Control, model	37.6	43.5	22.1	18.7
Non-paretic	25.8 (3.6)	28.7 (3.5)	16.7 (1.7)	14.7 (2.3)
Paretic	16.3 (2.0)	19.5 (2.6)	4.6 (1.0)	7.8 (1.1)
Paretic, model	16.5	21.6	6.8	9.2
NP/C ratio	0.65	0.74	0.86	0.88
P/NP ratio	0.63	0.68	0.28	0.53
P/C ratio	0.41	0.50	0.24	0.47
Male				
Control	66.4 (8.6)	66.8 (6.9)	38.2 (4.9)	33.6 (3.2)
Control, model	58.4	64.1	33.2	30.8
Non-paretic	57.7 (4.9)	59.1 (6.9)	35.8 (3.3)	32.0 (3.4)
Paretic	28.1 (2.9)	30.6 (5.0)	10.3 (2.4)	12.3 (2.2)
Paretic, model	26.2	31.7	11.0	13.9
NP/C ratio	0.87	0.89	0.94	0.95
P/NP ratio	0.49	0.52	0.29	0.38
P/C ratio	0.42	0.46	0.27	0.37
Combined				
Control	53.0 (5.8)	52.8 (5.4)	28.8 (3.8)	25.1 (3.0)
Non-paretic	41.8 (5.6)	43.9 (5.9)	26.3 (3.4)	23.4 (3.3)
Paretic	22.2 (2.1)	25.0 (3.0)	7.5 (1.5)	10.1 (1.0)
NP/C ratio	0.79	0.83	0.91	0.93
P/NP ratio	0.53	0.57	0.28	0.43
P/C ratio	0.42	0.47	0.26	0.40

Strength data presented as group mean (standard error), Nm. Model data presented as the single-DOF maximum torque output from the optimization (Nm). Ratios presented are of group averages

*AB* abduction,* AD* adduction, *ER* external rotation, *IR* internal rotation, *C* control, *NP* non-paretic,* P* paretic

**Fig. 3:**
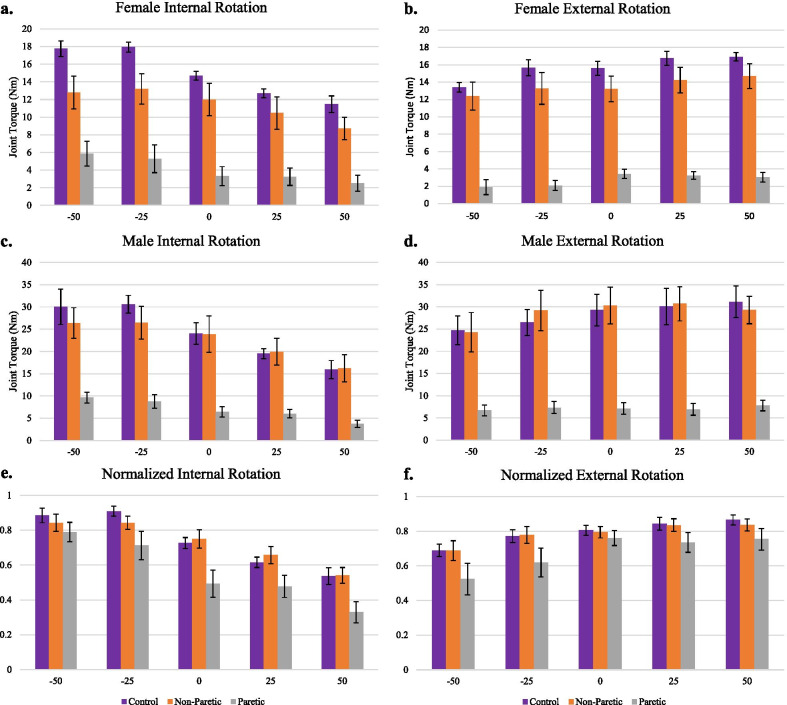
Dual-task performance. Bar plots with standard error of the isometric internal (**a**, **c**) and external (**b**, **d**) rotation torque generated under the different loading conditions for females (n = 6/group) and males (n = 6/group). Negative x-axis is % max adduction while positive x-axis is % max abduction and 0 indicating the unloaded or arm-weight fully supported condition. The bottom plots (**e**, **f**) depict the average internal and external strengths of the 12 participants in each group normalized to their maximal strengths

Dual-task results are presented in Fig. 3 as bar plots of the internal and external rotation strength at each load-level for each group (Fig. 3a–d presents actual joint torque, Fig. 3e–f presents joint torques *normalized* to each individual’s maximal voluntary torque in the corresponding direction). As shown in Fig. 3a–d and even more so in Fig. 3e–f, a common trend emerges across all groups: all limbs behave as the paretic arm was expected to—they are less able to generate out-of-synergy joint torques.

Table 2 shows the results of the four linear mixed-effects models on the normalized strength data. External rotation (ER) and internal rotation (IR) during adduction are listed first while external rotation and internal rotation during abduction are listed last.

**Table 2:** Linear Mixed Models for each of the four task combinations

	DF num	DF den	F-value	P-value
External Rotation during Adduction				
Group	2	46.91	5.96	0.0049
Load-Level	2	78.87	8.98	0.0003
Group*Load	4	78.87	0.85	0.4988
Internal Rotation during Adduction				
Group	2	51.5	8.96	0.00046
Load-Level	2	80.67	12.45	0.00002
Group*Load	4	80.67	1.2	0.32
External Rotation during Abduction				
Group	2	45.65	4.17	0.022
Load-Level	2	78.07	0.69	0.50
Group*Load	4	78.07	0.41	0.80
Internal Rotation during Abduction				
Group	2	49.01	18.32	0.00000
Load-Level	2	79.85	13.12	0.00001
Group*Load	4	79.85	0.35	0.84

Factors included Group (Control, Non-paretic, Paretic), Load-level (0, 25, 50%), and their interaction

*DF* degrees of freedom, *Num* numerator, *Den* denominator

As seen in Table 2, group was a significant main effect for each combination of adduction or abduction with external rotation or internal rotation. In each of the task combinations, paretic arms generated less torque (relative to their maximum) compared to controls and non-paretic arms, averaged across all load-levels (ER during Adduction: p = 0.0049, IR during Adduction: p = 0.00046, ER during Abduction: p = 0.022, IR during Abduction: p < 0.00000).

Load-level was significant for all task combinations except for external rotation during abduction (ER during Adduction: p = 0.0003, IR during Adduction: p = 0.00002, ER during Abduction: p = 0.503, IR during Abduction: p = 0.00001).

**Fig. 4:**
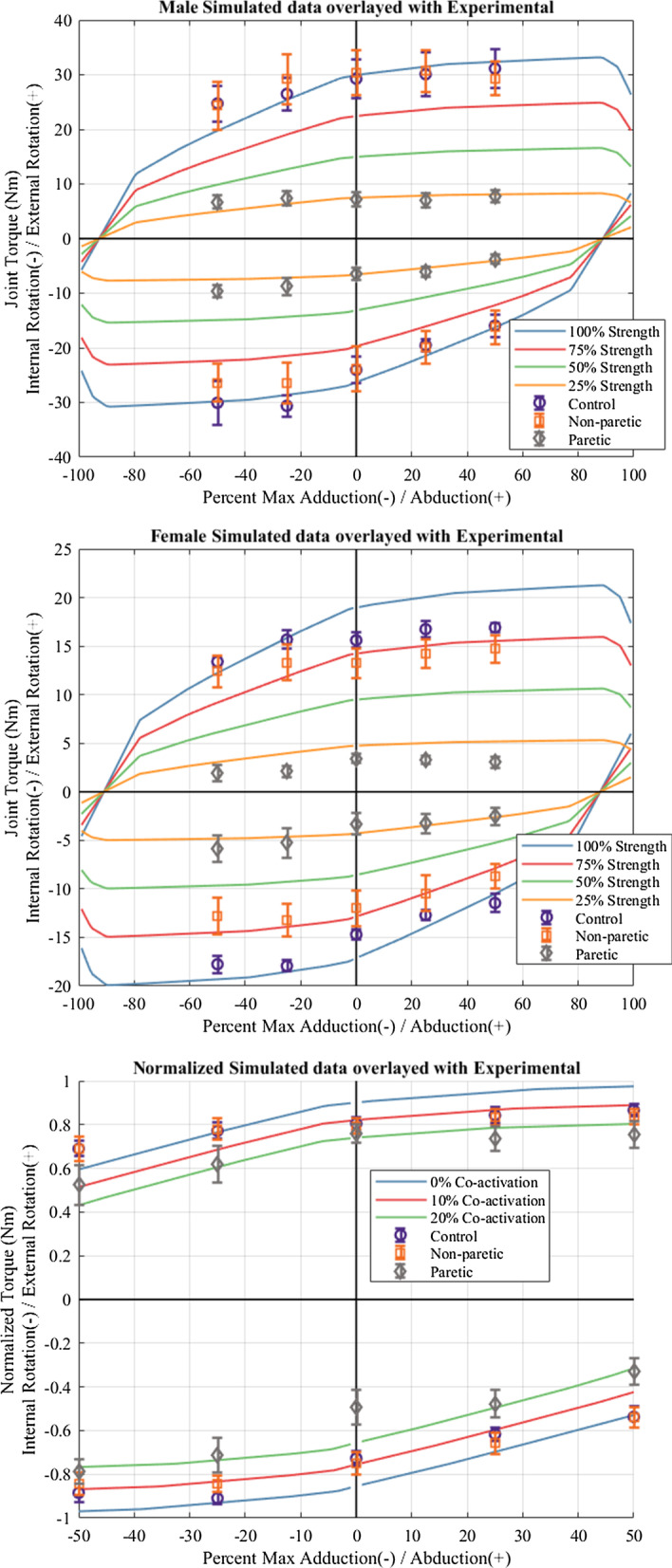
Modeled dual-task strength. Solid lines are simulated maximal joint torques using OpenSim setup with similar posture to the prescribed protocol. Experimental data overlayed with mean (± standard error). (Top) Females; (Middle) Males; (Bottom) All data normalized to maximum humeral rotation torque (external or internal). Background co-activation limits of 10 and 20% were added to simulate hypertonia

The group and load-level interaction was not significant in any task combination indicating that the differences in external and internal rotation strength across load-levels was not different between groups (their slopes are not different). As seen in both Figs. 3 and 4, the ability to generate external rotation torque increased with decreasing adduction, and the ability to generate internal rotation torque increased with decreasing abduction and with increasing adduction across all groups.

Figure 4 depicts the simulated joint torques across all load-levels, -100% (maximal adduction) to 100% (maximal abduction), overlaid by the experimental data for each group (*top—*females, *middle—*males, *bottom—*combined *normalized* strength). This includes a simulation of loss of muscle strength across all muscles of 25, 50, and 75% (*top* and *middle*) as well as a simulation of the effects of increased background activation (hypertonia) of 10 and 20% (*bottom*). The trends of the model match those of the experimental data. A simulated strength of 25% generated simulated torques that reflected paretic arm performance. Simulated co-activation levels of 10% generally followed the control and non-paretic arm data, while increased co-activation levels of 20% seemed to follow the paretic arm performance.

## Discussion

This study examined single-DOF internal/external rotation strength, and for the first time, combined internal/external rotation with abduction/adduction in individuals with and without stroke. Importantly, we attempted to better understand the factors contributing to the conventional inclusion of internal/external rotation in stroke-related stereotypical or synergy patterns of movement.

We hypothesized that the paretic arm would be less able to generate external rotation torque during adduction, less able to generate internal rotation torque during abduction, and conversely, more able to generate external rotation torque during abduction, and more able to generate internal rotation during adduction secondary to the elicitation of abnormal synergies. This was confirmed for all but external rotation during abduction in which there was no effect of load. There was also no effect of load in the external rotation-abduction condition for the control and non-paretic arm groups. The lack of significance may be due to fact that the external rotators (infraspinatus, teres minor) and the abductors (deltoid, supraspinatus) are different sets of muscles and are thus have more pure lines of action into their respective directions [25, 26, 31]. In contrast, the internal rotators and adductors are mostly the same sets of muscles thus creating torque in both directions simultaneously. Therefore, load would not affect the maximal amount of external rotation that could be generated during different amounts of abduction. This can be observed in the slope of both the experimental data as well as the simulated data (Fig. 4).

While three of four of these hypotheses were confirmed for the paretic arm, they were *also* confirmed for the control and non-paretic arms. There was no detected difference of the effect of load between groups. Most notably, all groups had limitations in “out-of-synergy” dual-tasks (internal rotation/abduction and external rotation/adduction). These results agree with recent results from Baillargeon et al. [16], in which the weakest torque directions in a young healthy population were combinations of abduction with internal rotation and adduction with external rotation, suggesting an alternative explanation for multi-DOF coupling in the glenohumeral joint.

The unexpected finding that the effect of load was not different between paretic arm and non-paretic or control arm performance suggests that the limited capacity to combine external rotation with adduction or internal rotation with abduction is a normal attribute of motor control. One explanation for this shared limitation is that there is a primary mechanical constraint that limits torque generation in these directions. We posit that biomechanical constraints due to muscle attachments and their corresponding actions naturally limit control of DOFs within the glenohumeral joint. Specifically, the primary adductors (latissimus dorsi, pectoralis major, and teres major) have moment arms with a strong component acting in both internal rotation as well as adduction [25, 26, 31]. Conversely, aside from the subscapularis (a more pure internal rotator), those same muscles are the primary internal rotators, with a significant component of the muscle pull acting in adduction. Thus, when a task requires adduction, the pectoralis major and latissimus dorsi are activated and intrinsically generate internal rotation torque, limiting the amount of net external rotation that can be generated. Conversely, when a task requires internal rotation, adduction torque will automatically be generated, opposing any attempted abduction torque generation. Thus, in a combined torque task where the abduction load must be controlled, the amount of internal rotation that can be generated will be limited by the amount of abduction that can offset or negate the biomechanically coupled adduction torque that occurs during internal rotation.

This shared biomechanical constraint was reflected in the musculoskeletal simulation. The simulation and experimental data for each sex, female data (Fig. 4, top), and male data (Fig. 4, middle), were similar, indicating that muscle actions, and more broadly, muscle biomechanics limit performance during this dual-task. Although the male data matches the model fairly well, the female data has some discrepancies in the external rotation direction, possibly due to the model being based on male anatomy. The model was built using bone and muscle size/length equivalent to a 50th percentile male, and while adjustments to muscle volume were made, adjustments to bone size, and moment arms were not. This may explain why the model fit to the male data is closer than the fit to the female data. Other limitations of the simulation include that it assumes intact/unimpaired motor control, cognition, and vision/perception—all of which may be impaired after stroke. While these impairments are mostly accounted for through inclusion/exclusion criteria as well as including testing of paretic, non-paretic, and control groups, they may partly explain differences between model output and study participant performance.

A second explanation for the shared limitation in capacity to combine internal rotation with abduction and external rotation with adduction between stroke and controls is that axial/proximal muscles are at least partially innervated by ipsilateral ventral corticospinal tracts. Following a stroke these tracts, used to drive the axial/proximal muscles of the affected side remain undamaged because they originate in the contralesional hemisphere. The non-significant interaction between load and group may then be explained by similar innervation of axial/proximal muscles regardless of the occurrence of stroke. However, we did observe a 50–75% loss of strength within the paretic arms suggesting a substantial role of the lateral corticospinal tract which is severely affected after stroke. With the loss of lateral corticospinal tract and presumed increased reliance on ventral corticospinal tract or other ipsilateral medial pathways, one would expect a different effect of load in the paretic arm. In the absence of this interaction effect of group and load, the explanation of ipsilateral medial motor pathways is less likely.

An important difference was observed between the performance of paretic arms compared to non-paretic and control arms. Paretic arms were weaker in single-DOF strength, but with the weakness accounted for by normalizing to their maximum, still demonstrated systematically lower performance (decreased normalized strength) in all four combined-torque conditions. Single-DOF weakness in the paretic shoulder is consistent with conventional stroke sequelae. And, our results appear generalizable since the magnitude of weakness observed in the present study is similar to prior work. Specifically, our data reflect previously reported baseline strength values for abduction (25 Nm), adduction (33 Nm), external rotation (8 Nm), and internal rotation (12 Nm) in a similar but larger cohort (N = 32) as part of a recent chronic stroke rehabilitation trial [32].

The systematically lower performance in the combined-torque tasks could reflect the presence of related stroke sequelae such as impaired cognition or motor planning, or hypertonia (generalized tonic co-activation). Impaired cognition and motor planning is less likely since performance was not different between the control and non-paretic arms, implying that participants with stroke were cognitively capable of completing the task in similar fashion to controls. Hypertonia that presents at rest and during tasks or movement after stroke [33–37] may better explain the systematic reduction in performance of paretic arms. This is supported by the simulations where minimal muscle activations were increased, which caused a drop in feasible maximal dual-task torque. As seen in Fig. 4 (bottom), increasing co-activation decreased dual-task performance across all load-levels and tasks, similar to what was seen in the experimental data. This additional constraint simulated increased global background activity or hypertonia. This would mean, for example, that during a maximal external rotation task, antagonist muscles such as the subscapularis, latissimus dorsi, and pectoralis major would also be activated, limiting the torque production in the desired (external rotation) direction. Loss of strength alone does not account for this drop in performance; once the modeled data simulating weakness are normalized to their maximum strength there is no observed drop in normalized dual-task performance (all simulated strength levels trace the blue line in the bottom of Fig. 4). Per the changes seen in this simulation, increased co-activation (hypertonia) may account for the observed group differences across all load-levels in this dual-task. We explored loss of strength and co-activation separately although there is an obvious interaction, as these effects were beyond the aims of this study. Further work will include intramuscular electromyography of the rotator cuff, possibly during sleep, to more accurately capture background muscular activity.

The extrapyramidal tracts, including the corticoreticulospinal and corticovestibulospinal tracts, implicated in control of posture and tone [38] and hypothesized to tune or modulate motor commands [37], are disinhibited [37] or upregulated [10, 39, 40] post stroke. Our results corroborate those findings in that hypertonia results from the increased activity of the extrapyramidal tracts, which relies on monoamines that in turn increase, more diffusely, the excitability of the remaining motor neuron pool. Further work is needed to separate the concurrent and related impairments of abnormal synergy and hypertonia that reflect an interaction between remaining ipsilesional corticofugal projections, upregulation of corticobulbospinal projections, and task-specific postural and tone requirements.

This study attempted to quantify the reported visual observations of abnormal synergy within humeral external and internal rotation, albeit in chronic stroke compared to acute and sub-acute stroke in which the original observations were made. These results do not support the inclusion of internal and external rotation within the definition of abnormal synergy. The similarity in effect of load between stroke and control suggests common biomechanical limitations in multi-DOF shoulder torque tasks due to musculoskeletal anatomy. These data, including the simulations, suggest an upregulation or increased activity of the extrapyramidal or tonal control system [10, 37, 39, 40], combined with severe strength impairments, especially in humeral external rotation, explaining the differences seen after stroke in this combined torque task.

The study design had some limitations. Despite significant main effects, the small sample size and large variance within the stroke population may have underpowered the statistical evaluation of an interaction effect of group and load. During the dual-task paradigm, we did not test at higher load-levels (> 50% abduction/adduction strength), which is known to maximize upper extremity synergy expression. While testing at higher loads becomes difficult secondary to motor control, fatigue, and discomfort, it may have indicated a minimal contribution of abnormal synergy to paretic arm performance that was not observed in the present study. Such an effect may be difficult to detect given the converging nature of the simulated dual-task strength curve at higher load-levels (decreased external rotation during adduction or decreased internal rotation during abduction). It is also possible that there was no group and load interaction because the task was proximal and difficult or effortful enough to fully activate the extrapyramidal system, thereby washing out the interaction effect expected of the paretic group across loads. This would implicate an effort- based effect versus a specific abduction load effect as previously proposed.

## Conclusion

This study examined humeral rotation strength under different abduction and adduction loads in an attempt to evaluate the underlying factors affecting control of the glenohumeral joint after stroke. With severe generalized stroke-related weakness accounted for, we conclude that a mechanical constraint (muscle action) is the primary factor affecting the ability to control multiple DOFs within the glenohumeral joint. This effect was common across all paretic, non-paretic, and control arms. A difference in load-dependent change (abnormal synergy) was not detected for participants with stroke compared to controls. However, persons with stroke showed systematic decreased dual-task performance across all loads that may be attributed to hypertonia, another sequela of stroke that results from increased activity of monoaminergic contralesional corticobulbospinal projections. Future rehabilitation interventions for these DOFs should focus both on building strength and reducing co-activation, which may be feasible through pharmacology, or possibly reducing effort.

## Data Availability

The datasets generated and analyzed during the current study are not yet publicly available as they are being used for other applications but are available from the corresponding author on reasonable request.

## Abbreviations

AB: Abduction
AD: Adduction
DOF: Degree of freedom
ER: External rotation
IR: Internal rotation
IRB: Institutional Review Board
NP: Non-paretic
P: Paretic

## References

[1] Benjamin EJ, Muntner P, Alonso A, Bittencourt MS, Callaway CW, Carson AP, Chamberlain AM, Chang AR, Cheng S, Das SR, et al. Heart disease and stroke statistics-2019 update: a report from the American Heart Association. Circulation 2019; CIR0000000000000659. 10.1161/CIR.0000000000000659 30700139

[2] Kwakkel G, Kollen BJ, Krebs HI (2008). Effects of robot-assisted therapy on upper limb recovery after stroke: a systematic review. Neurorehabil Neural Repair.

[3] Anderson CS, Linto J, Stewartwynne EG (1995). A population-based assessment of the impact and burden of caregiving for long-term stroke survivors. Stroke.

[4] Mussa-Ivaldi FA, Hogan N, Bizzi E (1985). Neural, mechanical, and geometric factors subserving arm posture in humans. J Neurosci.

[5] Twitchell TE (1951). The restoration of motor function following hemiplegia in man. Brain.

[6] Brunnstrom S (1966). Motor testing procedures in hemiplegia: based on sequential recovery stages. Phys Ther.

[7] Dewald JP, Sheshadri V, Dawson ML, Beer RF (2001). Upper-limb discoordination in hemiparetic stroke: implications for neurorehabilitation. Top Stroke Rehabil.

[8] O'Sullivan SB, Schmitz TJ (2007). Physical rehabilitation.

[9] Dewald JP, Beer RF (2001). Abnormal joint torque patterns in the paretic upper limb of subjects with hemiparesis. Muscle Nerve.

[10] McPherson JG, Chen A, Ellis MD, Yao J, Heckman CJ, Dewald JPA (2018). Progressive recruitment of contralesional cortico-reticulospinal pathways drives motor impairment post stroke. J Physiol.

[11] Ellis MD, Acosta AM, Yao J, Dewald JPA (2007). Position-dependent torque coupling and associated muscle activation in the hemiparetic upper extremity. Exp Brain Res.

[12] Sukal TM, Ellis MD, Dewald JPA (2007). Shoulder abduction-induced reductions in reaching work area following hemiparetic stroke: neuroscientific implications. Exp Brain Res.

[13] Ellis MD, Lan YY, Yao J, Dewald JPA (2016). Robotic quantification of upper extremity loss of independent joint control or flexion synergy in individuals with hemiparetic stroke: a review of paradigms addressing the effects of shoulder abduction loading. J Neuroeng Rehabil.

[14] Tombu M, Jolicoeur P (2003). A central capacity sharing model of dual-task performance. J Exp Psychol Hum Percept Perform.

[15] Miller LC, Dewald JP (2012). Involuntary paretic wrist/finger flexion forces and EMG increase with shoulder abduction load in individuals with chronic stroke. Clin Neurophysiol.

[16] Baillargeon EM, Ludvig D, Sohn MH, Nicolozakes CP, Seitz AL, Perreault EJ (2019). Experimentally quantifying the feasible torque space of the human shoulder. J Electromyogr Kinesiol.

[17] Beer RF, Ellis MD, Holubar BG, Dewald JPA (2007). Impact of gravity loading on post-stroke reaching and its relationship to weakness. Muscle Nerve.

[18] Twitchell TE (1951). The restoration of motor function following hemiplegia in man. Brain.

[19] Mehrholz J, Pohl M, Platz T, Kugler J, Elsner B (2015). Electromechanical and robot-assisted arm training for improving activities of daily living, arm function, and arm muscle strength after stroke. Cochrane Database Syst Rev.

[20] Beer RF, Given JD, Dewald JPA (1999). Task-dependent weakness at the elbow in patients with hemiparesis. Arch Phys Med Rehab.

[21] Delp SL, Anderson FC, Arnold AS, Loan P, Habib A, John CT, Guendelman E, Thelen DG (2007). OpenSim: open-source software to create and analyze dynamic simulations of movement. IEEE Trans Biomed Eng.

[22] Saul KR, Hu X, Goehler CM, Vidt ME, Daly M, Velisar A, Murray WM (2015). Benchmarking of dynamic simulation predictions in two software platforms using an upper limb musculoskeletal model. Comput Method Biomec.

[23] Sohn MH, McKay JL, Ting LH (2013). Defining feasible bounds on muscle activation in a redundant biomechanical task; practical implications of redundancy (vol 46, pg 1363, 2013). J Biomech.

[24] McKay JL, Burkholder TJ, Ting LH (2007). Biomechanical capabilities influence postural control strategies in the cat hindlimb. J Biomech.

[25] Kuechle DK, Newman SR, Itoi E, Niebur GL, Morrey BF, An KN (2000). The relevance of the moment arm of shoulder muscles with respect to axial rotation of the glenohumeral joint in four positions. Clin Biomech.

[26] Ackland DC, Pandy MG (2011). Moment Arms of the Shoulder Muscles during Axial Rotation. J Orthop Res.

[27] Holzbaur KRS, Murray WM, Gold GE, Delp SL (2007). Upper limb muscle volumes in adult subjects. J Biomech.

[28] Vidt ME, Daly M, Miller ME, Davis CC, Marsh AP, Saul KR (2012). Characterizing upper limb muscle volume and strength in older adults: a comparison with young adults. J Biomech.

[29] Janssen I, Heymsfield SB, Wang ZM, Ross R (2000). Skeletal muscle mass and distribution in 468 men and women aged 18–88 yr. J Appl Physiol (1985).

[30] Heyward VH, Johannesellis SM, Romer JF (1986). Gender differences in strength. Res Q Exercise Sport.

[31] Ackland DC, Pak P, Richardson M, Pandy MG (2008). Moment arms of the muscles crossing the anatomical shoulder. J Anat.

[32] Ellis MD, Carmona C, Drogos J, Dewald JPA (2018). Progressive abduction loading therapy with horizontal-plane viscous resistance targeting weakness and flexion synergy to treat upper limb function in chronic hemiparetic stroke: a randomized clinical trial. Front Neurol.

[33] Knutsson E, Richards C (1979). Different types of disturbed motor control in gait of hemiparetic patients. Brain.

[34] Chae J, Yang G, Park BK, Labatia I (2002). Muscle weakness and cocontraction in upper limb hemiparesis: relationship to motor impairment and physical disability. Neurorehabil Neural Repair.

[35] Levin MF, Selles RW, Verheul MH, Meijer OG (2000). Deficits in the coordination of agonist and antagonist muscles in stroke patients: implications for normal motor control. Brain Res.

[36] Stoeckmann TM, Sullivan KJ, Scheidt RA (2009). Elastic, viscous, and mass load effects on poststroke muscle recruitment and co-contraction during reaching: a pilot study. Phys Ther.

[37] Li S, Chen YT, Francisco GE, Zhou P, Rymer WZ (2019). A unifying pathophysiological account for post-stroke spasticity and disordered motor control. Front Neurol.

[38] Lee J, Muzio MR. Neuroanatomy, extrapyramidal system. In: StatPearls. StatPearls Publishing; 2021. 32119429

[39] Ellis MD, Drogos J, Carmona C, Keller T, Dewald JPA (2012). Neck rotation modulates flexion synergy torques, indicating an ipsilateral reticulospinal source for impairment in stroke. J Neurophysiol.

[40] McPherson JG, Ellis MD, Heckman CJ, Dewald JP (2008). Evidence for increased activation of persistent inward currents in individuals with chronic hemiparetic stroke. J Neurophysiol.

